# Plasticity of *Phymatotrichopsis omnivora* infection strategies is dependent on host and nonhost plant responses

**DOI:** 10.1111/pce.13721

**Published:** 2020-02-14

**Authors:** Prasanna Kankanala, Piet Jones, Raja Sekhar Nandety, Daniel A. Jacobson, Kirankumar S. Mysore

**Affiliations:** ^1^ Noble Research Institute LLC Ardmore Oklahoma; ^2^ Biosciences Division Oak Ridge National Laboratory Oak Ridge Tennessee; ^3^ Bredesen Center for Interdisciplinary Studies University of Tennessee Knoxville Knoxville Tennessee

**Keywords:** fungal plasticity, hemi‐biotrophy, necrotrophy, nonhost resistance, Phymatotrichopsis root rot, plant–fungal interactions, RNA sequencing, systems biology

## Abstract

Necrotrophic fungi constitute the largest group of plant fungal pathogens that cause heavy crop losses worldwide. *Phymatotrichopsis omnivora* is a broad host, soil‐borne necrotrophic fungal pathogen that infects over 2,000 dicotyledonous plants. The molecular basis of such broad host range is unknown. We conducted cell biology and transcriptomic studies in *Medicago truncatula* (susceptible), *Brachypodium distachyon* (resistant/nonhost), and *Arabidopsis thaliana* (partially resistant) to understand *P. omnivora* virulence mechanisms. We performed defence gene analysis, gene enrichments, and correlational network studies during key infection stages. We identified that *P. omnivora* infects the susceptible plant as a traditional necrotroph. However, it infects the partially resistant plant as a hemi‐biotroph triggering salicylic acid‐mediated defence pathways in the plant. Further, the infection strategy in partially resistant plants is determined by the host responses during early infection stages. Mutant analyses in *A. thaliana* established the role of small peptides PEP1 and PEP2 in defence against *P. omnivora*. The resistant/nonhost *B. distachyon* triggered stress responses involving sugars and aromatic acids. *Bdwat1* mutant analysis identified the role of cell walls in defence. This is the first report that describes the plasticity in infection strategies of *P. omnivora* providing insights into broad host range.

## Abstract 2

*Phymatotrichopsis omnivora* can amend its infection strategy between necrotrophy and hemi‐biotrophy in dicotyledonous plants based on host defences, which indicates the basis of its broad host range.

## 1 INTRODUCTION

Plant pathogens are categorized based on their mode of acquiring nutrition (Laluk & Mengiste, 2010). Biotrophic pathogens derive nutrition from live plant cells, whereas necrotrophic pathogens derive nutrition from dead or dying plant cells. The hemi‐biotrophs have a transient biotrophic phase before becoming necrotrophs. Necrotrophic fungal pathogens are either host specific or broad spectrum. Broad‐spectrum necrotrophic fungal pathogens have myriad strategies to infect plant species including production of enzymes, toxins, and effectors, thus causing heavy crop losses annually (Laluk & Mengiste, 2010). *Phymatotrichopsis omnivora* (Duggar) Hennebert (G. M. Watkins & Watkins, 1940) is one such broad‐spectrum, filamentous, soil‐borne, necrotrophic pathogen that causes the destructive Phymatotrichopsis root rot (PRR) disease in Southwest USA and Northern Mexico (Uppalapati et al., 2010). This facultative saprophytic fungus becomes pathogenic during the dry summer months (Rogers, 1942). Penetration may occur through wounds or by mechanical action on the periderm of the roots in the field (Peltier, King, & Sampson, 1926). The typical disease symptoms include chlorosis, rapid wilting, and plant death. *P. omnivora* infects over 2,000 dicotyledonous plants but cannot infect monocotyledonous plants (Streets, 1937). Several members of Brassicaceae have been reported to escape PRR disease when grown as winter crops (Streets, 1937). *P. omnivora* causes severe disease in fibre and forage crops like cotton and alfalfa (*Medicago sativa*), respectively (Lyda, 1978). Farmers in Southern Oklahoma and Texas are reluctant to grow alfalfa in spite of its high economic and nutritional value due to persistence of PRR disease in this region. Since the first report of this disease in late 1800s (Pammel, 1888), no resistant cultivars have been identified. The molecular mechanisms of pathogen virulence, host susceptibility, and broad host range are not yet understood (Uppalapati et al., 2009).

In response to pathogen attack, plants are armoured with a two‐layered immune system. The initial defence relies on identifying pathogen‐associated molecular patterns (PAMPs). This recognition triggers plant immune responses termed as PAMP‐triggered immunity (PTI; Dodds & Rathjen, 2010). Damage‐associated molecular patterns (DAMPs) also trigger PTI with plant cell‐derived molecules due to tissue damage or trauma (Matzinger, 2002). The second type of defence relies on recognition of the pathogen avirulence proteins or effector proteins by plant resistance (R) proteins and is referred to as effector‐triggered immunity or ETI (Dodds & Rathjen, 2010). R proteins are comprised of many classes and subclasses based on the type of domains they encode, the majority being the nucleotide‐binding site–leucine rich‐repeats (NBS–LRR) class. The NBS–LRR class that contains Toll/interleukin‐1 receptor protein domain (TIR) at their N‐terminus are called as TIR–NBS–LRRs (TNLs), and the NBS–LRR class that contains coiled‐coil domain at the N‐terminus are labelled as coiled coin–NBS–LRRs (CNLs; Meyers, Kozik, Griego, Kuang, & Michelmore, 2003). Many of the TNL proteins interact with plant pathogens recognizing their effector proteins and triggering ETI (Macho & Zipfel, 2015; Thomma, Nurnberger, & Joosten, 2011). A subclass of R proteins that lack C‐terminal LRR domains (TN proteins) or NBS‐LRR domains (TX proteins) play a role in plant basal defence mechanisms that is dependent on *EDS1* gene and salicylic acid (SA)‐mediated plant defence pathways (Nandety et al., 2013). Similarly, several pathways involving proteins like NDR1, mitogen‐activated protein kinases, protein kinases, and brassinosteroid insensitive 1‐associated kinase 1 (BAK1) are implicated in plant defence responses to pathogens (Century, Holub, & Staskawicz, 1995; Genenncher et al., 2016; Song et al., 1995; Yasuda, Okada, & Saijo, 2017). Further, plants respond to biotrophic pathogens by triggering SA‐mediated defences and to necrotrophic pathogens by triggering ethylene (ET)‐ and jasmonic acid (JA)‐mediated defences (Browse, 2009; van Loon, Geraats, & Linthorst, 2006; Vijayan, Shockey, Lévesque, Cook, & Browse, 1998; Vlot, Dempsey, & Klessig, 2009). Thus, dissecting the host immune responses provides insight into pathogen infection strategies.

Extensive attempts to identify *P*. *omnivora*‐resistant *M. truncatula* or *M. sativa* genotypes were unsuccessful. Thus, to further understand the *P*. *omnivora*–host or *P*. *omnivora*–nonhost interactions and the broad host range of *P*. *omnivora*, *A. thaliana* and *B. distachyon* were included in this study. We performed comparative cell biology and transcriptional profiling of susceptible (*M. truncatula*), partially resistant (*Arabidopsis thaliana*), and resistant (nonhost; *Brachypodium distachyon*) interactions during *P*. *omnivora* infection. We found that *P*. *omnivora* exhibits high fungal plasticity and can infect plants either as a necrotrophic pathogen or as a hemi‐biotrophic pathogen. This plasticity provides insights into its broad host range. We also report two distinct plant stress responses when challenged with *P*. *omnivora*.

## 2 MATERIALS AND METHODS

### 2.1 Plant growth, fungal growth, and infection assays

The plants were grown in culture tubes as described previously (Uppalapati et al., 2009) with the following changes. *M. truncatula* (A17) seeds were scarified with sandpaper. *B. distachyon* (Bd21‐3) seeds were dehusked and surface sterilized by vortexing in 30% bleach solution for 7 min. *A. thaliana* (col 0) seeds were sterilized in 95% ethanol for 2 min, 30% bleach for 5 min, and followed by four washes in distilled water. *A. thaliana* seeds were stratified for 2 days at 4°C. *P. omnivora* isolate NFPO01 was isolated from infected alfalfa roots at Ardmore, OK in summer 2014 and cultured as described previously (Uppalapati et al., 2009). Four‐week‐old seedlings were infected with *P. omnivora* with wheat seed inoculum as described previously (Uppalapati et al., 2009).

### 2.2 Light, confocal, and scanning electron microscopy

Infected roots were stained with 10‐μg/ml wheat germ agglutinin coupled to green fluorescent dye Alexa Fluor 488 (WGA Alexa Fluor 488; Invitrogen Corp., Carlsbad, CA, USA), which was dissolved in distilled water for 30 min. The roots' cell walls were counterstained with 10‐μg/ml propidium iodide dissolved in distilled water for 15 min. Imaging was done as described previously (Ray, Guo, Kolape, & Craven, 2017). Roots from at least three different infected plants were studied for cell biology features at each infection time point. An average of 150 cells per root were analysed in each plant species. For *A. thaliana*, 15 different infected roots were observed at each infection time point. Epifluorescence microscopy images were captured on Zeiss Apotome 2 with Zen blue software. For scanning electron microscopy, infected roots were flash frozen and imaged with Hibachi tabletop scanning electron microscope (TM3030).

### 2.3 Transcriptional profiling and RT‐qPCR

Infected roots at 0, 1, 3, 5, 7, and 10 days post infection (dpi) were frozen in liquid nitrogen followed by homogenization with a mortar and pestle. RNA extraction was done with the Spectrum™ plant total RNA kit (Sigma‐Aldrich, cat # STRN50). *M. truncatula* roots of individual plants were harvested for each replicate. *A. thaliana* and *B. distachyon* roots of three individual plants were pooled for each replicate. Three replicates were sampled for each plant species. RNA samples were treated with deoxyribonuclease 1 (Invitrogen Corp). RNA sequencing libraries were prepared using the TruSeq Stranded mRNA Sample Preparation kits following the manufacturer guidelines (Illumina cat no. RS‐122‐2001). Individual libraries were uniquely indexed using TruSeq Single Indexes (Illumina cat no. RS‐122‐2002) and pooled in equimolar ratio. The pooled libraries were sequenced on a NextSeq 500 Sequencing system (Illumina, CA, USA). RT‐qPCRs were done as described previously (Gill et al., 2018) with the primer sets listed in supporting information Table S1.

### 2.4 Bioinformatic analysis pipeline for transcriptional data

The reference genome for *A. thaliana* was obtained from Phytozome (https://phytozome.jgi.doe.gov/pz/portal.html; Lamesch et al., 2012). The 18 samples of paired‐end reads were assessed for quality using FastQC (Simon, 2010) and trimmed using Cutadapt (Marcel, 2011). Count data was then obtained using Kallisto (kmer length = 31; Bray, Pimentel, Melsted, & Pachter, 2016). To improve the confidence in the read quantification, we performed 1,000 bootstrapped samples and took the average as our transcript quantification. Expression count for each respective plant species were analysed after trimmed mean of *M* values normalization (Robinson, McCarthy, & Smyth, 2010). This generated a matrix with samples as the columns and transcripts as the rows. Alignment and read statistics are provided in Table S2. The estimated count data was then processed using VOOM (Law, Chen, Shi, & Smyth, 2014) for the purposes of differential analysis using the R package LIMMA (Smyth, 2005). Multiple hypotheses correction was performed using the Benjamini–Hochberg procedure, with a false discovery rate cut‐off of 0.01 (Benjamini & Hochberg, 1995). For differential comparisons, we compared the transcript counts of the time points post infection with the time 0 control. In addition, differential comparisons were also performed between consecutive time points post infection.

Gene ontology enrichments were done using AgriGO (Tian et al., 2017) for the three plant interactions. Pathway enrichments and protein domain enrichments in *M. truncatula* were performed using MedicMine (Krishnakumar et al., 2015) and in *A. thaliana* were performed using ThaleMine (Rosen et al., 2014). The co‐expression analysis was performed on the genes that are statistically significantly different compared with genes in control sample (0 dpi). The expression values were grouped into early stage (1 and 3 dpi) and late stage (7 and 10 dpi), respectively. For each of the respective early‐stage/late‐stage data sets, an all‐pairs Pearson correlation coefficient calculation of the differential transcripts was performed. Gene pairs with positive correlation above 0.80 or a negative correlation below −0.75 were further analysed as putatively co‐expressed genes. Gene enrichment analysis was performed on the early‐stage/late‐stage categorized normalized gene counts using the right‐tailed Fisher exact test, accounting for multiple hypotheses bias (false discovery rate < 0.05). Further enrichment analyses were performed based on culture tube and cell biology observations overlapping specific time points post infection, as given in Table 1. Mercator (Lohse et al., 2014) was used to annotate the reference transcripts and obtain respective Mapman descriptive terms. The co‐expression clusters, differential expression results, along with the Mapman annotations were all visualized in Cytoscape 3.6 (Shannon et al., 2003).

**Table 1:** Gene enrichment analysis corresponding to infection biology of *Phymatotrichopsis omnivora*

Infection stage	Cell biology features	Transcriptional processes
*Medicago truncatula* early infection stage	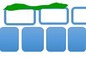 Mycelia grows over the root	Monooxygenases *O*‐methyltransferases
*Brachypodium distachyon* early infection stage	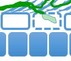 Root induce root hair formation Restricted intracellular and intercellular pathogen growth	Enhanced cellular carbohydrate metabolism Organic acid synthesis
*Arabidopsis thaliana* early infection stage	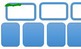 Restricted fungal growth	Cellular detoxification Toxin metabolism Defence response to fungi
*M. truncatula* late infection stage	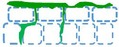 Intercellular fungal growth Cortical penetration	Downregulation of peptidases, kinases, LRRs. proteolytic and hydrolytic activities
*B. distachyon* late infection stage	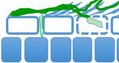 Extensive root hair formation Failed pathogen penetration into cortex	Enhanced cellular carbohydrate metabolism Cellular detoxification processes Aromatic acid synthesis
*A. thaliana* late infection stage	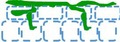 Intracellular growth in 20% of roots	Defence responses Cellular detoxification processes

Similar pipelines were used to analyse *B. distachyon* and *M. truncatula* transcriptome data. The reference genome for *B. distachyon* (*B. distachyon* v3.1) was downloaded from Phytozome. For the *B. distachyon* co‐expression analysis, a threshold of 0.95 and −0.90 was applied to the positive and negative correlation values, respectively. The *M. truncatula* expression data were analysed using the Mt4.0v1 (Tang et al., 2014) reference genome from Phytozome. Co‐expression analysis threshold of 0.98 and −0.95 was applied to the positive and negative correlation values, respectively.

A further combined analysis of all the expression count matrices of the plant species was performed, after collectively normalizing the raw expression count data from the plant species using trimmed mean of *M* values normalization (Robinson et al., 2010). An all‐pairs Pearson correlation coefficient calculation was then performed. Gene pairs that had an absolute correlation value greater than .98 were then clustered using Markov clustering (Enright, Van Dongen, & Ouzounis, 2002), with an inflation value of 7.

## 3 RESULTS

### 3.1 *M. truncatula*, *B. distachyon*, and *A. thaliana* respond differently to *P. omnivora* infection in culture tube assays

We used a previously established culture tube‐based disease assay system (Uppalapati et al., 2009) with three model plant species, *M. truncatula* (susceptible), *B. distachyon* (nonhost/resistant), and *A. thaliana* (partially resistant). In the susceptible *M. truncatula*, the fungal mycelia grew into the culture media tubes from 1 dpi onwards leading to wilting by 10 dpi and chlorosis and death by 18 dpi (Figure S1). The fungal growth features were similar in the resistant *B. distachyon* culture tubes except that the plants did not wilt and die. Although *B. distachyon* plants were stressed by 18 dpi with yellow leaves, they survived for 8 weeks (Figure S1). Interestingly, *P. omnivora* growth was inhibited in *A. thaliana* at 1 dpi. The mycelia grew slowly by 3 dpi into the agar, avoiding roots when possible. The fungal growth was active at 5 dpi, still avoiding the roots when possible. By 10 dpi, the *A. thaliana* plants exhibited leaf yellowing and died by 18 dpi (Figure S1). Dark pigmentation was seen in the agar at the root–shoot junctions in all the three plant species between 5 and 7 dpi when the mycelia grew to the bottom of the tubes, potentially increasing the nutrient competition between plant and fungus.

### 3.2 *P. omnivora* grows as a necrotrophic pathogen in susceptible *M. truncatula*

**Figure 1:**
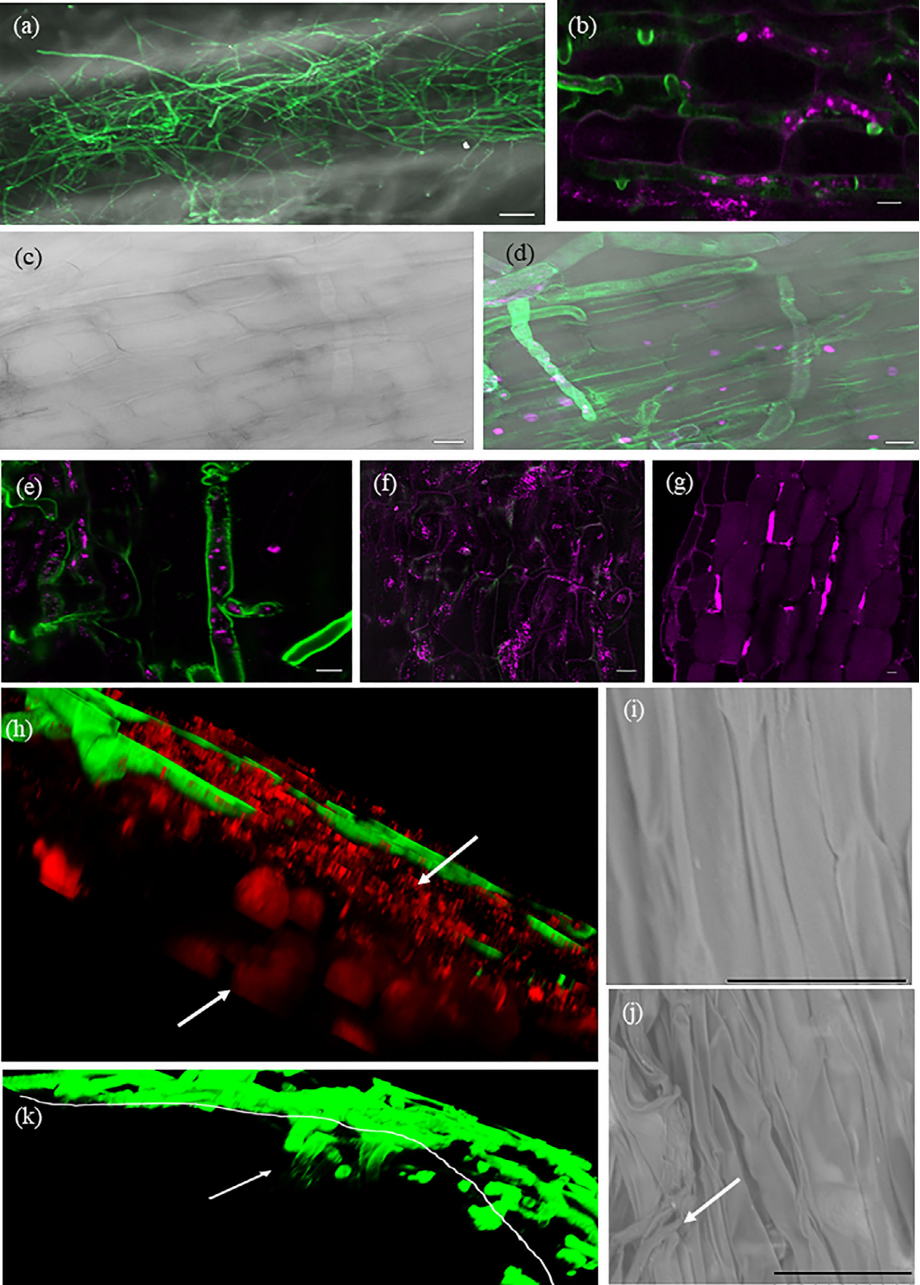
Biology of *Medicago truncatula* roots infected with *Phymatotrichopsis omnivora*. The fungus was stained with wheat germ agglutinin Alexa 488 indicated as green colour. (a) Epifluorescence image of whole root image at 7 days post infection (dpi) captured with Zeiss Apotome 2, scale 100 μm; (b,e–h,k) confocal images. (b) Infected root epidermal cell walls stained with propidium iodide indicated as magenta colour with intercellular fungal hyphae (green) at 5 dpi. (c) Bright field image of 7 dpi epidermal cells at the infection site. (d) Epifluorescence image of root epidermal cells in (c) 7 dpi epidermal cells at the infection site when treated with propidium iodide stained the nuclei (shown in magenta), indicating the cells are either dead or dying. (e–g) *M. truncatula* with apoplast tagged with mCherry seen as magenta colour at 6 dpi. (e) Very little to no magenta colour in the infected epidermal cells; (f) distorted apoplast marker at the leading edge of infection; (g) intact apoplast away from the infection; (h) 3D projection of 32 z‐stack images captured at 1.5‐μm intervals of infected *M. truncatula* endoplasmic reticulum tagged with mCherry at 3 dpi and plasmolyzed with 0.75‐M sucrose. The top arrow points to distorted epidermal endoplasmic reticulum (shown in red), and the bottom arrow points to the intact endoplasmic reticulum in cells beneath the epidermis indicated by plasmolysis. (b–h**)** Scale bar 10 μm. (i–j) Scanning electron microscope images of uninfected and infected epidermal cells at 6 dpi; scale bar 30 μm. White arrow indicates hyphae. (k) 3D projection of 20 z‐stack images captured at 1.5‐μm intervals of infected roots at 7 dpi. The white line demarcates the epidermal cell layer. The arrow points to the hyphae penetrating into the cortex layers

Live cell confocal microscopy was used to study the infection process of *P. omnivora*. Three hundred root epidermal cells per root were examined during this interaction. The fungal mycelia grew as a mantle over *M. truncatula* roots until 5 dpi as reported earlier (Figure 1a; Uppalapati et al., 2009). Intercellular epidermal growth was observed sporadically at this stage (Figure 1b). Propidium iodide (PI)‐stained nuclei of 90% of epidermal cells underneath the mycelial mantle and at the growing ends of the hyphae indicating the plant cells in *M. truncatula* were damaged (Figure 1c, d). To further characterize infection features, transgenic *M. truncatula* expressing fluorescent mCherry‐tagged apoplast‐localized protein (Ivanov & Harrison, 2014) was infected with *P. omnivora*. These transgenic plants showed little fluorescence in the region of the root epidermis where the fungal mycelia were growing, indicating apoplast damage (Figure 1e). The fluorescence in the uninfected cells at the leading edge of infection was distorted (Figure 1f), whereas the fluorescence of the apoplast marker, 15 to 20 cells away from the infection site, was intact (Figure 1g). To test the integrity of the membranes in the cells surrounding the infected regions, *M. truncatula* line expressing mCherry‐tagged endoplasmic reticulum (ER)‐localized protein (Ivanov & Harrison, 2014) was infected with *P. omnivora*. The infected roots when plasmolyzed revealed that the surface epidermal cells had collapsed ER, whereas the layers beneath had intact ER (Figure 1h). Scanning electron microscope imaging of the infected and uninfected roots further affirmed the epidermal cell damage in infected roots (Figure 1i,j). *P. omnivora* attempted intercellular penetration into the cortex between 5 and 7 dpi (Figure 1k). These observations indicated that *P. omnivora* grew intercellularly in *M. truncatula* root epidermal cells, collapsing the cells ahead of its infection in a typical necrotrophic strategy.

### 3.3 *P. omnivora* growth is effectively inhibited in the resistant *B. distachyon* roots

**Figure 2:**
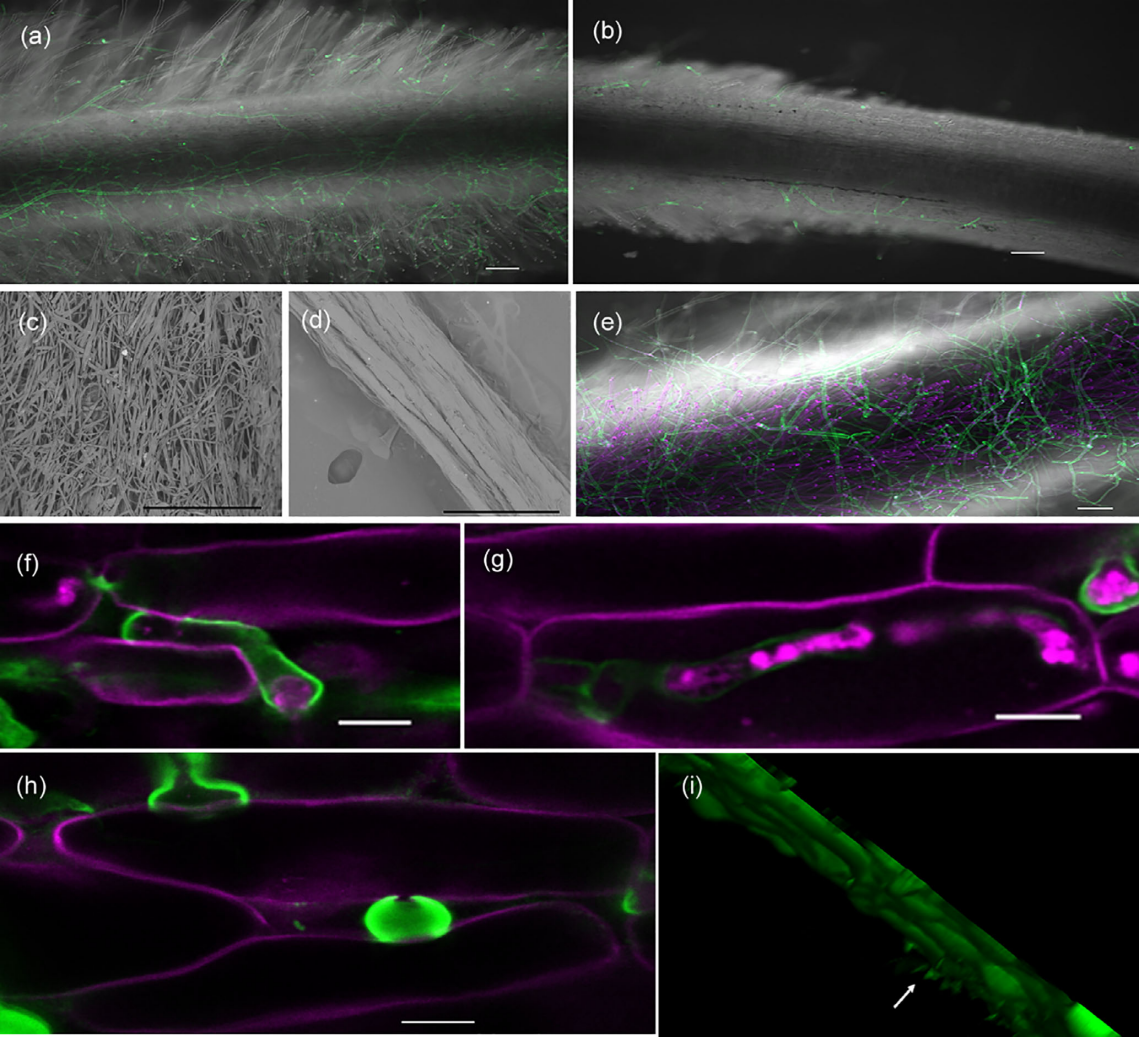
Biology of *Brachypodium distachyon* roots infected with *Phymatotrichopsis omnivora*. (a–b,e) Epifluorescence images of infected whole root at 4 days post infection (dpi); scale bar 100 μm. (a) Profuse root hair growth where the fungal hyphae grow along the roots. (b) Lack of root hairs at the leading end of the fungal hyphae; (c) scanning electron microscope image of infected root at 5 dpi; (d) scanning electron microscope image of uninfected root at 5 dpi; (c–d) Scale bar 300 μm. (e) Epifluorescence image of infected root stained with propidium iodide (magenta) and wheat germ agglutinin Alexa 488 (green); scale bar 100 μm. Magenta indicates root hairs, and green indicates fungal hyphae. (f–i) Confocal images of infected roots; scale bar 10 μm. (f) Intercellular hyphal growth in between epidermal cells at 3 dpi; (g) intracellular hyphae that was contained in the root epidermal cell at 3 dpi; (h) intercellular hyphae penetrating through the epidermal cells, widening the gaps between cells indicated at 4 dpi. (i) 3D projection of 17 z‐stack images captured at 1.5‐μm intervals at 7 dpi. Arrow indicates the penetration pegs that fail to grow further

Strikingly, in the resistant/nonhost *B. distachyon*, the roots induced active root hair growth along the root surface where the *P. omnivora* mycelia grew (Figure 2a). There were none or very few root hairs at the leading edge of mycelia growth on the roots (Figure 2b). In scanning electron microscope, the profuse root hair growth in the infected roots obscured the view of the root epidermal cells (Figure 2c), whereas the epidermal cells in uninfected regions of the roots were visible (Figure 2d). PI staining indicated that *P. omnivora* mycelia were intertwined in the root hairs, which restricted access to the epidermal cells (Figure 2e). However, in some instances, the mycelia grew between the root hairs, and intercellular epidermal growth was similar to *M. truncatula*. Three hundred epidermal root cells were examined per root and intercellular epidermal growth was observed in 7% to 10% of the epidermal cells (Figure 2f). Unlike in *M. truncatula* roots, *P. omnivora* also attempted intracellular growth in 5% of epidermal cells in the roots that were observed. However, these intracellular hyphae did not grow well and were restricted to the infected cell (Figure 2g). The fungus also attempted intercellular cortical penetration similar to *M. truncatula* around 7 dpi but was not successful. The hyphae pushed forward through the cell walls into the cortical layers (Figure 2h) that appeared as small penetration peg‐like structures (Figure 2i), which were also restricted. Thus, *B. distachyon* employed various strategies to successfully inhibit fungal infection.

### 3.4 *P. omnivora* grows intracellularly in the partially resistant *A. thaliana* roots

**Figure 3:**
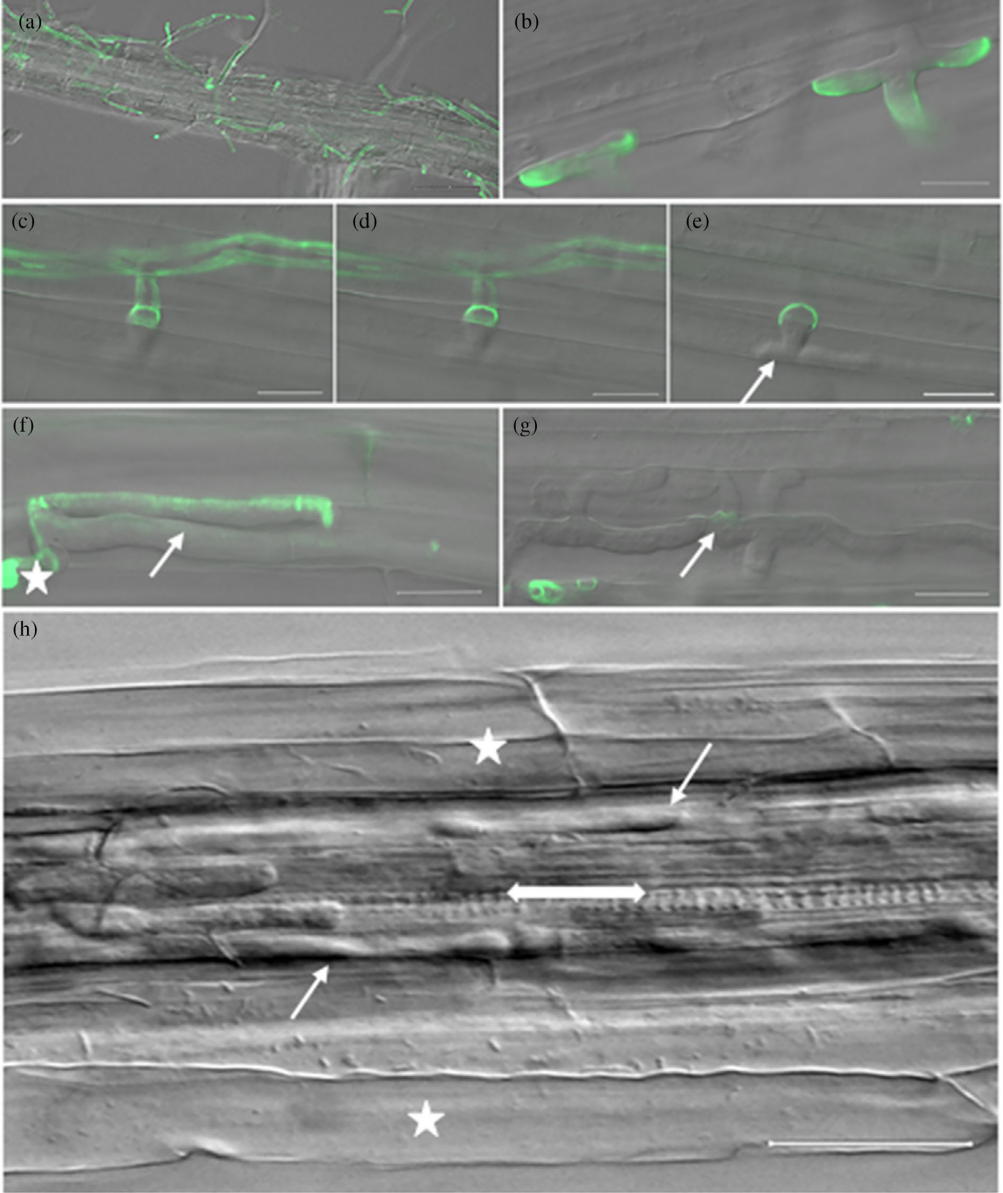
*Arabidopsis thaliana* roots infected with *Phymatotrichopsis omnivora*. (a) Epifluorescence image infected whole roots at 4 days post infection (dpi); scale bar 100 μm. (b–h) Epifluorescence images of infected roots from 2 to 5dpi; scale bar 10 μm. (b) Fungal hyphae appressed to the root epidermal wall just before penetration at 2 dpi; (c–e) serial images of hyphae at penetration site. The hyphae fail to stain with wheat germ agglutinin Alexa 488 when they enter into the root epidermal cell indicated with (e) arrow. (f–g) Arrows indicate the wheat germ agglutinin Alexa 488 staining pattern in intracellular hyphae. Star indicates intracellular hyphal branching following penetration; (h) intracellular hyphae (indicated by single‐pointed arrows) growing close to vascular regions (indicated by double‐pointed arrow) in the root away from the infection site at 5 dpi. Hyphal growth was not seen in epidermal cells, which is indicated by stars in these regions

*P. omnivora* growth was sparse in culture tubes along the *A. thaliana* roots, compared with *M. truncatula* and *B. distachyon* (Figure 3a). The hyphae grew slowly between 2 to 4 dpi and penetrated intracellularly into the epidermal cells. Intercellular growth was not observed in this interaction. By 10 dpi, 15% to 20% of the total roots observed were infected. An average of 10 intracellular infection sites per root in a total of 15 roots were examined. No specialized structures were observed at penetration sites (Figure 3b). *P*. *omnivora* hyphae that grew on the root surface were stained with the WGA Alexa 488 stain, but the intracellular penetration hyphae did not bind the stain, indicating differential staining properties (Figure 3c–e). To facilitate efficient uptake of stain into the roots, the staining solution was vacuum infiltrated into the infected roots for 20 min followed by 1‐hr incubation at room temperature. The stain was usually observed in the first penetrated hyphal cell and at the junctions where it moves from one cell to another (Figure 3f,g). The first penetrated hyphae are generally divided into two branches, one of which grows more robustly (Figure 3e,f). After penetration, the intracellular hyphae tend to grow along the vascular tissues in the infected root. We observed the hyphal growth along the vascular cells in the roots, which is further away from the penetration sites (Figure 3h **)**. The active *P. omnivora* growth in the culture tube assays for *A. thaliana* corresponds to the intracellular epidermal growth (Table S3). These results indicate that *A. thaliana* efficiently blocks *P. omnivora* growth initially, but the fungus eventually penetrates the cells and grows intracellularly. Taken together, these data indicate that *P. omnivora* employs two different infection strategies in the susceptible and partially resistant plants. Further, the difference in infection biology in the resistant and partially resistant plants (Figure S2) also suggests differential host perception and defence responses.

### 3.5 Transcriptional analysis indicated the infection process as a gradual step‐up phenomenon

**Figure 4:**
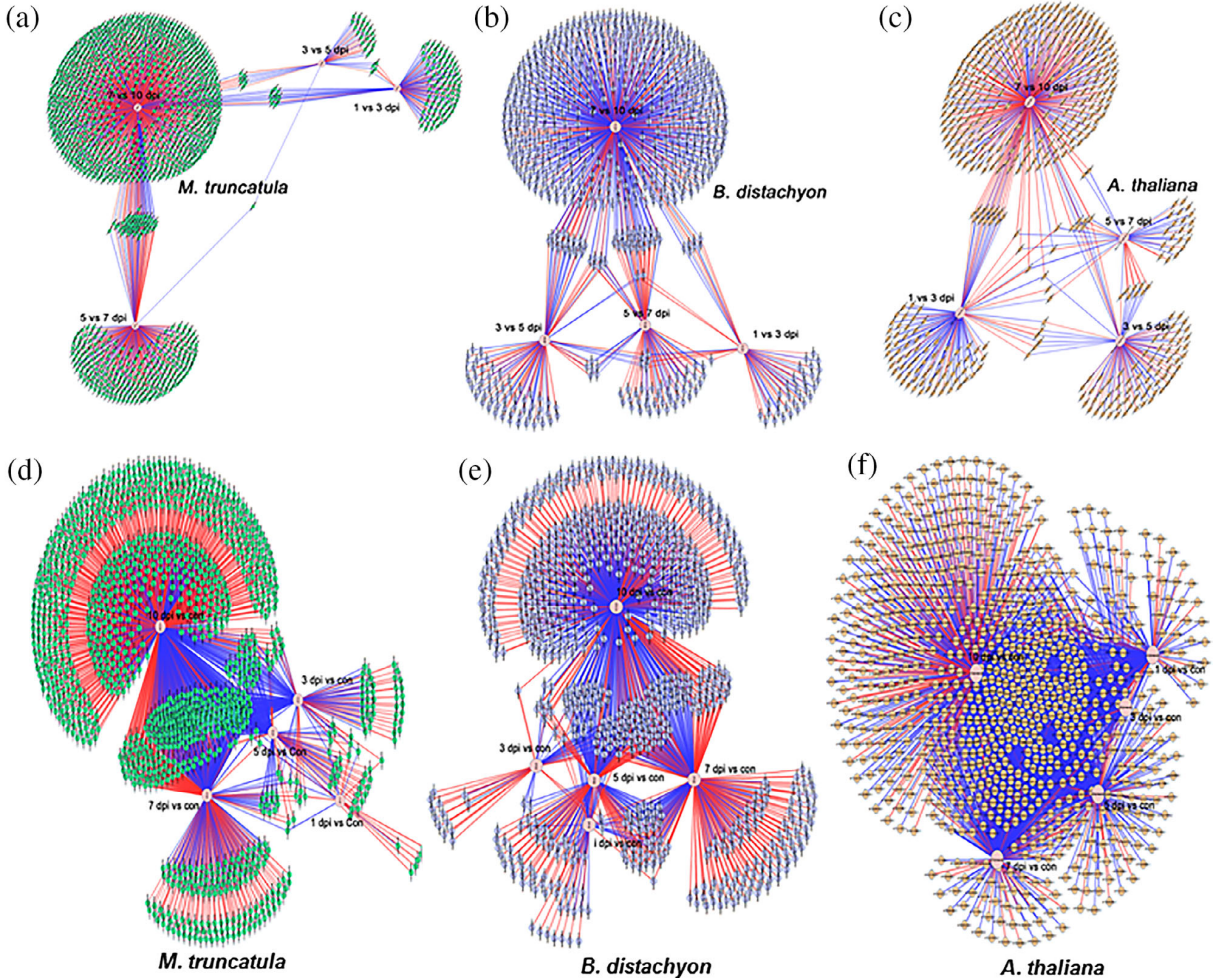
Biological networks of differentially expressed genes (DEGs). The networks were generated in cytoscape 3.6.1. (a–c) DEGs of adjacent infection time points; (d–f) DEGs of each infection time point compared with control (0 days post infection). Red lines indicate downregulated genes, and blue lines indicate upregulated genes. The nodes of the networks indicate the time points used for comparison. The green edges represent *Medicago truncatula* DEGs; blue edges represent *Brachypodium distachyon* DEGs; and yellow edges represent *Arabidopsis thaliana* DEGs. Supporting information [Link], [Link], and S3 were used to generate these cytoscape networks

To determine plant molecular responses of all three species tested to *P. omnivora* infection, RNA sequencing was conducted in the three interactions at six infection timepoints—0, 1, 3, 5, 7, and 10 dpi. The gene expression analyses of each time point were compared with either 0 dpi (control) and/or adjacent time points. Differentially expressed gene (DEG) networks comparing adjacent time points identified very few common genes during disease progression in the three plant species (Figure 4a–c), indicating that the disease progression is a gradual step‐up process in all the interactions. DEG networks comparing each infection time point with control (0 dpi) identified several common genes during disease progression (Figure 4d–f). The transcriptional activity at 7 and 10 dpi had enhanced DEGs in all the interactions perhaps due to the enhanced nutrient stress from 7 dpi when the fungus grows to the bottom of the culture tube. The DEGs also indicate that there is enhanced downregulation of genes in *M. truncatula* (red lines) and enhanced upregulation of genes in *B. distachyon* (blue lines) during *P. omnivora* infection.

Further analysis of DEGs indicated a shift in the molecular response in disease progression after 5 dpi in *M. truncatula*. The number of downregulated DEGs increased, and upregulated DEGs decreased from 7 dpi onwards (Table S4). In the *B. distachyon*, the number of upregulated and downregulated DEGs were similar at all time points. The partially resistant *A. thaliana* exhibited enhanced transcriptional activity at 1 dpi and had consistently higher numbers of upregulated DEGs throughout the infection process (Table S4).

### 3.6 Gene ontology analysis in the three interactions identified mechanisms of disease susceptibility and disease resistance

In the susceptible *M. truncatula*, there were no significant gene ontology (GO) enrichments at 1 dpi (Table S5a–b). The plant launched active defences between 3 and 5 dpi indicated by upregulation of genes in the phenylpropanoid pathway and detoxification processes. A significant shift occurred in the molecular responses from 7 dpi onwards, and genes involved in defence response like protein kinases, LRR proteins, and peptidases were repressed. Several genes involved in cellular detoxification, chloroplast, secondary metabolism, and sugar metabolism were downregulated, suggesting a compatible response (Table S5a–b).

In the resistant/nonhost *B. distachyon*, cellular carbohydrate metabolism processes and genes involved in cellular detoxification like peroxidases and monoxygenases were upregulated from 1 dpi onwards and sustained throughout the infection process. At 3 dpi, protein kinase *SD‐2b* family genes, *LRK10L‐2* subfamily genes, and genes involved in lignan synthesis, which play a role in plant defence, were upregulated. At 7 and 10 dpi, genes encoding aldolase, chorismates, steroids, and other aromatic amino acids were significantly upregulated (Table S6a–b).

GO enrichments in partially resistant *A. thaliana* indicated that the plant launches an active defence response from 1 dpi onwards (Table S7a–b). Biotic stress response genes involved in production of small heat shock proteins (sHSPs), chitin recognition, phenylpropanoid pathway, glutathione *S*‐transferases, indole‐containing compounds, and toxin metabolism were induced from 1 dpi onwards. Wounding‐induced JA‐responsive genes like lipoxygenase (*LOX3*), and 12‐oxophytodienoate (*OPR2*) were also induced by 1 dpi. Genes involved in production of glucosinolates, alkaloids and stilbenoids/gingerols, cell wall synthesis, and lignin metabolism and several genes involved in SA signalling were also upregulated by 3 dpi. At 10 dpi, a distinct GO category, “killing cells of other organisms,” that contained a large number of genes belonging to the defensin‐like family and the scorpion toxin‐like knottin super family were induced. A large number of *WRKY* transcription factor genes were induced throughout the interaction, which were absent in the other plant species.

**Figure 5:**
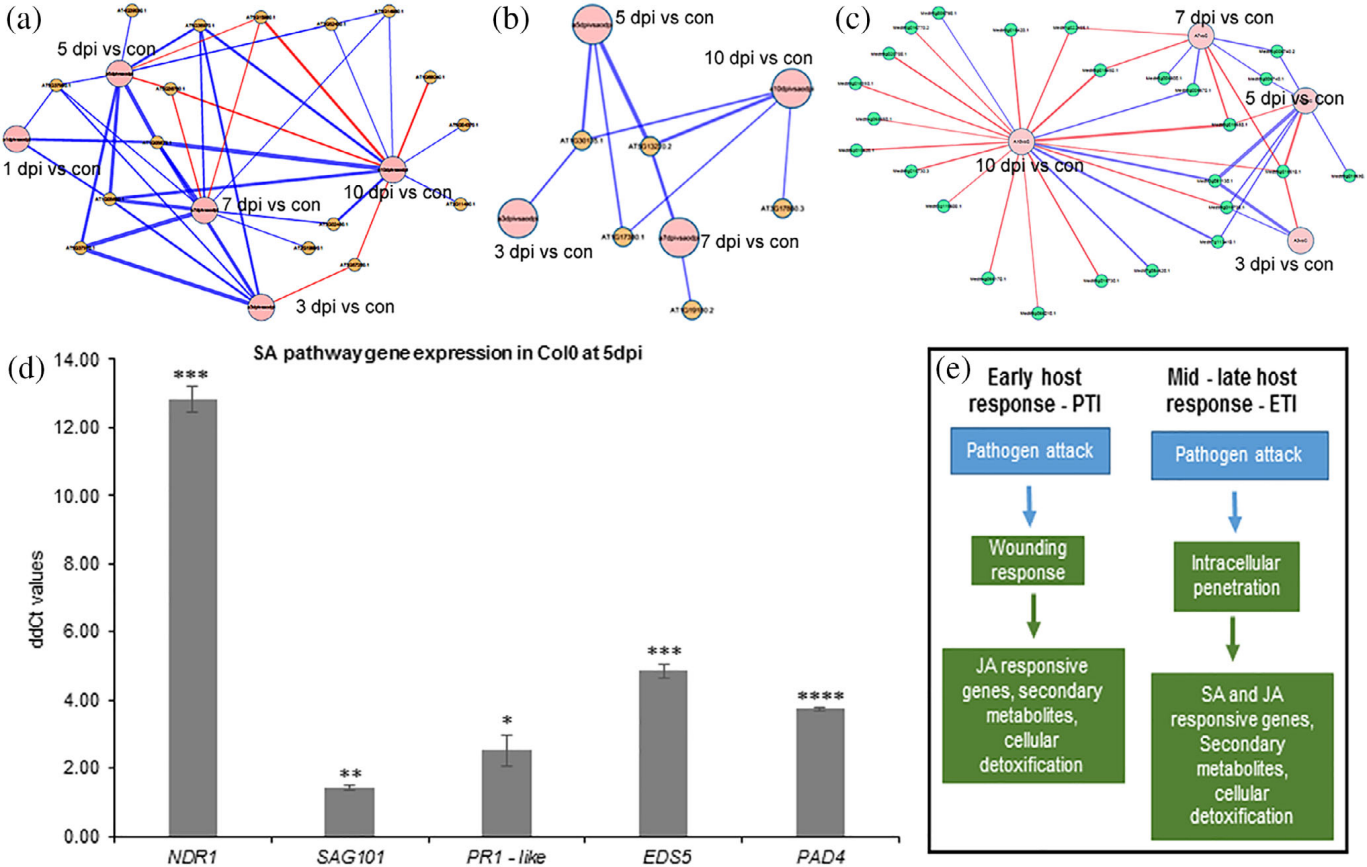
Analysis of SA and JA pathway genes during infection process. (a–c)Biological networks generated in cytoscape 3.6.1. Red lines indicate downregulated genes, and blue lines indicate upregulated genes. The thickness of the lines corresponds to the degree of differential expression. The nodes indicate the time points used for comparison. These networks were generated by selecting for respective pathway genes from the networks in Figure 4d–f. (a) Network of differentially expressed SA‐dependent pathway genes in *Arabidopsis thaliana* at different infection time points compared with control. (b) Network of differentially expressed *JAZ* genes in *A. thaliana* at different infection time points. (c) Network of differentially expressed genes in SA‐dependent pathway in *Medicago truncatula* at different infection time points compared with control. (d) RT‐qPCRs of *A. thaliana* SA—defense signalling pathway genes, *NDR1*, *SAG101*, *PAD4*, *PR1* like, and *EDS5* at 5 days post infection. Comparison of means was done using Student's *t*‐test^.*^ *p* < .05, ^**^ *p* < .01, ^***^ *p* < .01, ^****^ *p* < .0001. (e) Flow chart mapping the PTI and ETI responses in *A. thaliana*. ETI, effector triggered immunity; JA, jasmonic acid; PTI, pathogen‐associated molecular patterns‐triggered immunity; SA, salicylic acid

In *A. thaliana*, SA biosynthetic and SA‐responsive gene induction corresponded to the *P. omnivora* growth into the medium from 3 dpi onwards in culture tube assays (Figure 5a). Several Jasmonate–ZIM domain (*JAZ*) repressor genes were induced during this time that repress the JA‐mediated biotic defence responses (Figure 5b). Similar analysis in *M. truncatula* and *B. distachyon* interactions did not identify any SA biosynthetic or SA‐responsive gene induction. In *M. truncatula*, wounding‐induced JA‐responsive genes like *LOX* genes were induced at 3 and 5 dpi but were later repressed (Figures 5c and **S3**). In *A. thaliana*, SA‐responsive genes like *PAD4*, *EDS5*, *PR1* like, and *SAG101* were highly induced (Figure 5d). In summary, the gene expression pattern in *A. thaliana* indicates initial pathogen perception and wound‐triggered responses as part of the PTI responses at 1 dpi. After 3 dpi, SA‐mediated defence signalling was induced corresponding to intracellular growth, indicating ETI responses (Figure 5e).

**Figure 6:**
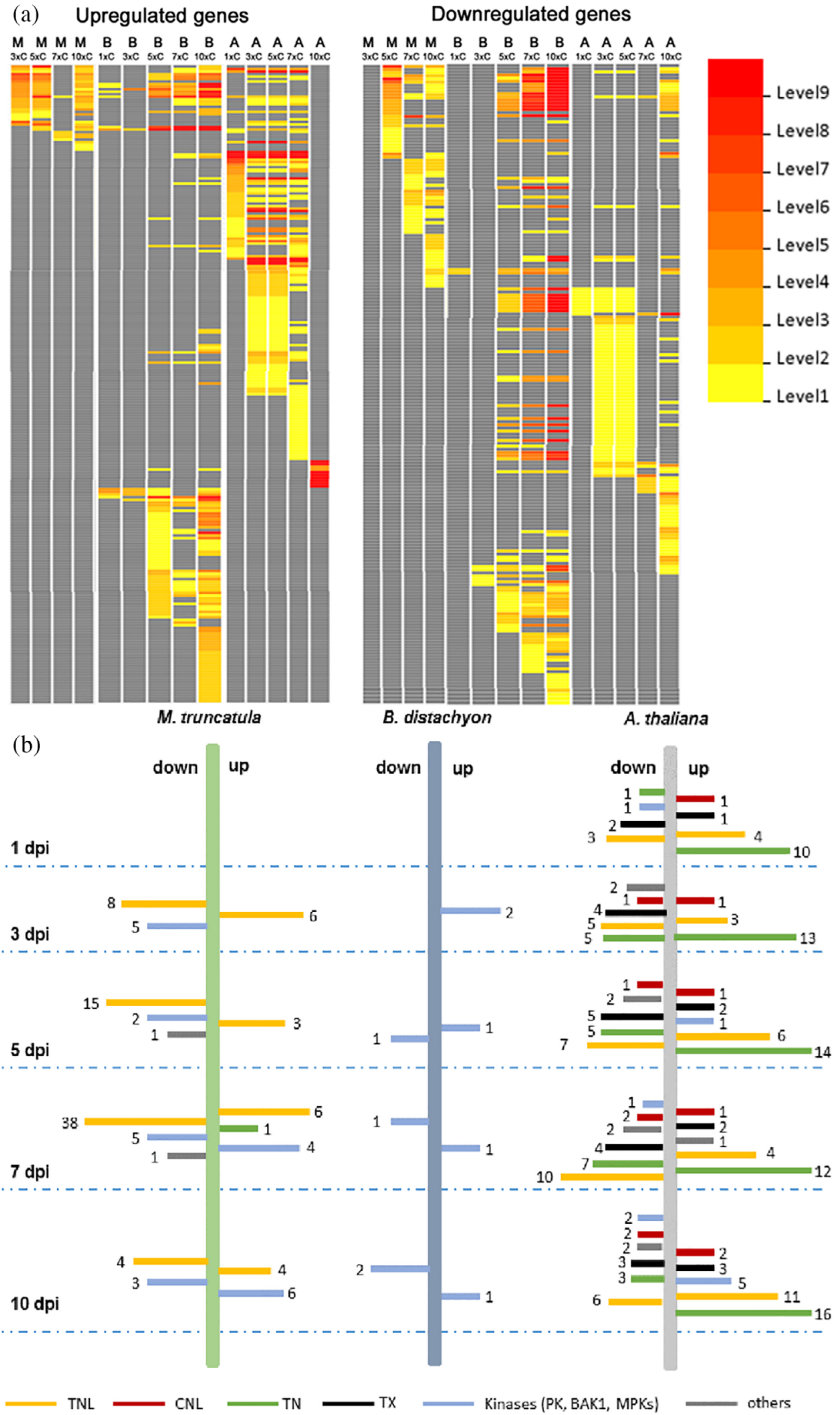
Differentially expressed gene (DEG) analysis across three plant species and defence‐related gene analysis. (a) Comparative gene ontology enrichment of upregulated and downregulated DEGs across three plant species. Scale bar indicates increasing significance levels from 1 to 9. The details of these gene ontology classes are listed in Table S8a–b. (b) Defence‐related gene analysis in the upregulated and downregulated DEGs in three plant species. Further details of these genes are listed in Table S11

We further compared the GO enrichments of DEGs between the three plant species using AgriGO to identify common GO enrichments in the three interactions. The common GO categories in the three plant species included genes involved in processes like ion homeostasis, oxidative stress response, and response to chemical stimulus. Distinct sets of genes were induced and repressed at different infection time points in the *B. distachyon* and *A. thaliana* (Figure 6a; Table S8).

### 3.7 Gene enrichments at early and late infection time points indicated distinct molecular processes were induced in the three plant species

We did gene enrichments corresponding to early (1 and 3 dpi) and late (7 and 10 dpi) infection time points to identify key plant processes. The early and late infection stages also correspond to key cell biology events during infection (Table 1). In the susceptible *M. truncatula*, the early infection stage was characterized by plant defences that were repressed at late infection stage when the fungus penetrates into the cortex (Tables 1 and S9). During early infection stage in the resistant *B. distachyon*, active root hair growth and occasional intercellular or intracellular epidermal growth was observed when genes involved in cellular carbohydrate metabolism, signalling kinases, and organic acid synthetic processes were enriched. The late infection stage corresponded to the successful blockage of pathogen penetration into the cortex when genes involved in cellular detoxification and aromatic acid production were further enriched (Tables 1 and S9).

In the partially resistant *A. thaliana*, unique disease‐responsive genes were induced at 1, 3, 7, and 10 dpi (Tables 1, and S9). Response to heat, light, and wounding were unique responses in *A. thaliana* and predominantly observed from 1 to 7 dpi. Gene enrichment analysis at early infection stage in *A. thaliana* identified genes involved in defences against fungal pathogens, cellular detoxification processes, and secondary metabolite production. During this early infection stage, the fungal growth is largely inhibited by the plant. This indicates that the plant is actively defending against the pathogen as early as 1 dpi. The late infection stage is characterized by intracellular growth in some root cells when the defence responses are further enhanced (Tables 1 and S9). These data indicate that the three plant species tested have different responses to *P. omnivora*. To further understand the defence responses, we analysed defence‐related genes in the three plant species.

### 3.8 Nonhost and partially resistant plants induce two distinct stress response pathways against *P. omnivora*

We examined the expression of genes that encode proteins involved in plant immunity summarized in Table S10 in the three interactions. At 1 dpi, there were no defence‐related DEGs in *M. truncatula* and *B. distachyon*, whereas *A. thaliana* upregulated 16 defence‐related DEGs. These included TNL, TN, TX, and CNL classes of R genes (Figure 6b). The differential expression of CNLs were unique to *A. thaliana* interaction. The majority of the TNLs, with a few exceptions, were highly repressed in *M. truncatula*. Several Pto‐like protein kinase‐encoding genes, with roles in plant defence, were upregulated in *A. thaliana* and repressed in *B. distachyon* and *M. truncatula* (Table S11). The *B. distachyon* interaction was characterized with the consistent upregulation of a BAK1 homolog (*Bradi4g18280*) from 3 to 10 dpi (Figure 6b). This analysis further confirms that the defence responses induced by *B. distachyon* and *A. thaliana* against *P. omnivora* are distinct from each other.

To identify the components of biotic defence pathways in these two interactions, we conducted Mapman analysis of DEGs at each infection time point. The resistant *B. distachyon* analysis did not induce any ETI‐mediated responses with the exception of a few defence genes (Figure S4). The predominant response in this plant species was mediated through abiotic stress response that included germin‐like protein encoding genes. Auxin and ET biosynthetic genes and cellular detoxification genes encoding proteins such as glutathione synthases, peroxidases, ubiquitin E3 RING proteins, and HSPs were induced throughout the disease progression. The partially resistant *A. thaliana* plants induced ETI‐mediated responses as early as 1 dpi. SA, JA, and ET responses were observed from 1 dpi onwards (Figure S4). The signalling gene *At5g64890* was induced from 3 dpi onwards and encodes for the PROPEP2 small peptide. PROPEP2 is processed into PEP2 and binds to PepR2 DAMP receptor that triggers defence responses (Figure S4). This analysis further confirmed that the two plants induced different defences.

### 3.9 Correlation networks and hub genes determined diverse functional pathways in *A. thaliana* and *B. distachyon*

**Figure 7:**
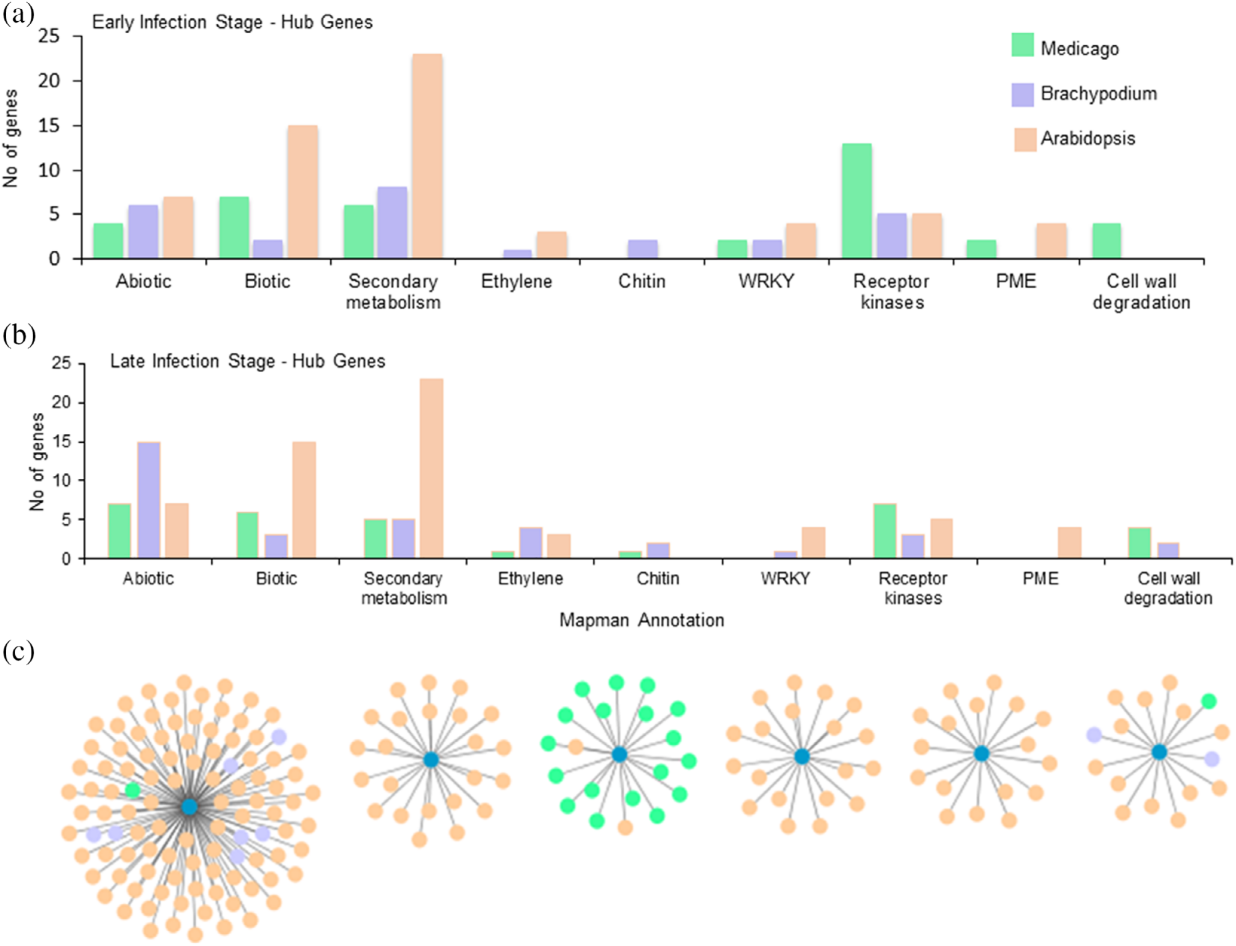
Correlation network data analysis. Graphs (a,b) indicate the number of hub genes in functional gene categories in twofold and above upregulated differentially expressed genes in the three plant species at early (1 and 3 days post infection [dpi] compared with control) and late infection stages (7 and 10 dpi compared with control), respectively. (c) Interspecies correlation networks clusters of differentially upregulated genes at 5 dpi. Orange edges indicate *Arabidopsis thaliana* genes, purple edges indicate *Brachypodium distachyon* genes, and green edges indicate *Medicago truncatula* genes. The three plant species formed separate clusters indicating absence of interspecies correlations. Datasets [Link], [Link] were used for correlation network analysis

To characterize the functional pathways during plant responses to *P. omnivora*, we generated correlation networks for twofold and above upregulated DEGs in the early and late infection stages. The top 10% of genes with highest centrality in the correlation networks are identified as hub genes that are hypothesized to play significant roles in the infection process (Albert, Jeong, & Barabási, 2000; Langfelder, Mischel, & Horvath, 2013). Mapman annotation was used to identify functional classes of these hub genes. *M. truncatula* had both abiotic and biotic stress response genes, secondary metabolism genes, signalling receptor kinases, pectin methyl esterases (PMEs), and cell wall degradation genes upregulated at the early infection stage (1–3 dpi). By the late infection stage (7–10 dpi), the genes encoding signalling receptor kinases, secondary metabolism, and PMEs were downregulated (Figure 7a–b). The resistant *B. distachyon* plants had enhanced abiotic stress response genes. Wounding‐induced ET‐responsive genes and chitinase genes were induced in *B. distachyon*, indicating fungal recognition at early infection stage. Secondary metabolism genes, *WRKY* genes, and signalling receptor kinase‐encoding genes were also induced, indicating an active stress response. The late infection stage was characterized by enhanced abiotic stress‐responsive genes and genes encoding alkaloids.

The hub genes in partially resistant *A. thaliana* plants included higher numbers of biotic stress response genes and secondary metabolite genes in both early and late infection stages. PME‐encoding genes were expressed in both early and late infection stages (Figure 7a–b). The biotic stress response in *A. thaliana* included genes encoding TNL proteins, chitinases, a serine‐type endopeptidase inhibitor, a defensin‐like protein, transmembrane proteins, two respiratory burst proteins (AtRBOHD and AtRBOHC), and small peptides (PROPEP1 and PROPEP3). Secondary metabolite genes in the glucosinolate pathway, lignin synthesis, chalcone synthesis, flavonol production, and alkaloid production were included in the hub genes for *P. omnivora*–*A. thaliana* interaction. *WRKY33*, *WRKY40*, and *WRKY53* that were previously reported in pathogen response were also upregulated.

To identify common functional pathways involved in the three plant responses, we generated cross‐species co‐regulatory networks at each infection time point. The resulting network clusters were separated by species with very little to no cross‐species regulatory genes (Figure 7c). In summary, the transcriptional analysis identified that *B. distachyon* perceives fungal presence at early infection stages and induces immune response potentially triggered by wound‐induced ethylene signalling pathway. *A. thaliana* also perceives fungal presence at early stage and induces DAMP‐mediated PTI response followed by SA‐mediated ETI defence responses.

### 3.10 Mutant analysis identified PEP1 and PEP2 in conferring PTI‐mediated resistance in *A. thaliana* against *P. omnivora* infection

**Figure 8:**
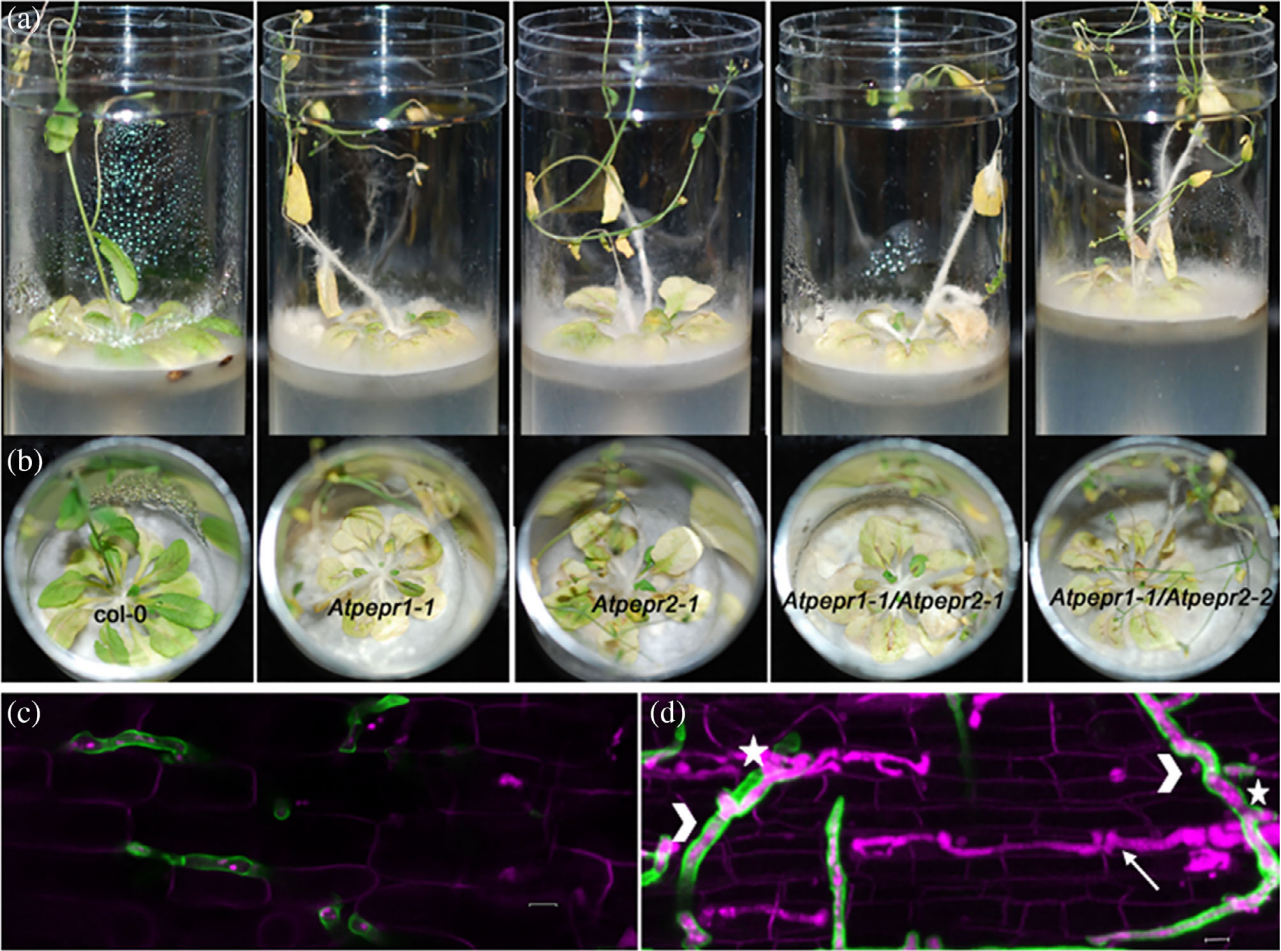
*Arabidopsis thaliana* and *Brachypodium distachyon* mutant analyses. (a,b) At 12 days post infection, the *Atpepr1*, *Atpepr2*, and double mutants were hypersusceptible to *Phymatotrichopsis omnivora* infection compared with Col‐0 wild‐type plants. Panel (b) is the aerial view of the culture tubes in panel (a). (c,d) Confocal images of *B. distachyon* roots infected with *P. omnivora* at 4 days post infection roots. Scale bars: 10 μm. (c) Wild‐type BD21‐3 roots infected with *P. omnivora* stained with wheat germ agglutinin Alexa 488 (green) and propidium iodide (magenta). The fungal hyphae grow intercellularly, indicated by the green colour. (d) Collapsed image of 17 z‐stack optical sections imaged at 1.5‐μm thickness of *Bdwat1* mutant root infected with *P. omnivora*. Stars indicate penetration point into epidermal cells. Arrow indicates intracellular hyphae growing from cell to cell. The arrow heads point to the hyphae growing on the surface of the root that is stained with wheat germ agglutinin Alexa 488 in green coloured

Transcriptional analysis identified *PROPEP1* and *PROPEP2* genes involved in *A. thaliana* immune response. PEP1 and PEP2 are small peptides derived from the C‐terminal of PROPEP1 and PROPEP2, respectively. They bind to PEPR1 and PEPR2 receptors, respectively, and activate the DAMP signalling pathway (Yamaguchi, Huffaker, Bryan, Tax, & Ryan, 2010). To determine the role of PROPEP1 and PROPEP2 in providing resistance against *P. omnivora*, we obtained *Atpepr1‐1* (SALK id CS800015), *Atpepr2‐1* (SALK id CS800008) null mutants, and *Atpepr1‐1 Atpepr2* double mutants in two different *Atpepr2* allelic backgrounds used in a previous study (Yamaguchi et al., 2010). Culture tube infection assay indicated that all the mutants were hypersusceptible to *P. omnivora* compared with the wild‐type control plants (Figure 8a,b). The mutant plants died 5 days earlier than the wild‐type plants confirming that PEP‐mediated immune responses in *A. thaliana* play a role in conferring resistance against *P. omnivora*.

### 3.11 *B. distachyon* cell wall mutants were partially compromised in resistance allowing hemi‐biotrophic fungal infection

Because *P. omnivora* fails to infect monocotyledonous, we hypothesized that the fungal arsenal is incompatible with intact monocot root cell walls. To test this hypothesis, we used a *B. distachyon* cell wall mutant for infection assay. Walls are thin 1 (WAT1) is a tonoplast protein that is involved in normal secondary cell walls in *Zinnia elegans*, *A. thaliana*, and *B. distachyon* (Hsia et al., 2017; Pesquet et al., 2005; Ranocha et al., 2013). *Bdwat1* mutants are developmentally delayed with irregular xylem walls, irregular cell shapes, and stunted growth (Hsia et al., 2017). Culture tube infection assays were done with homozygous *Bdwat1* mutants. The wild‐type plants induced root hair formation as described above. Occasional intercellular penetration (10%) and intracellular penetration (3%) of the root epidermal cells were observed (Figure 8c). Unlike wild‐type plants, the *Bdwat1* mutants failed to induce root hair formation upon infection. Intracellular penetration was observed in 80% of the epidermal cells in four different roots by 4 dpi. *P. omnivora* grew in the first penetrated cell, packed it with bulbous hyphae (Figure 8d), and then grew as thin, long hyphae penetrating from cell to cell (Figure 8d). The intracellular hyphae did not bind the WGA Alexa 488 stain similar to *A. thaliana* infection. Although *P*. *omnivora* penetrated epidermal cells successfully, no cortical penetration was observed, indicating that the mutant is only partially susceptible.

## 4 DISCUSSION

We conducted a comprehensive cell biology and transcriptional analysis of *P. omnivora* interactions in three different plant species: *M. truncatula*, *B. distachyon*, and *A. thaliana*. The stronger virulence of the pathogen in *M. truncatula* was supported by the fact that *P. omnivora* caused typical disease symptoms like mycelial mantle formation, wilting, and chlorosis as described previously (Uppalapati et al., 2009; G. M. Watkins, 1938; G. M. a. W. Watkins, M.O., 1940). In previous studies, *M. truncatula* defence responses were induced by 3 dpi but returned to basal levels by 5 dpi (Uppalapati et al., 2009). In this study, the responses diminished by 7 dpi. This difference could be attributed to different isolates used in this study. This repression of plant defences corresponded with *P. omnivora* penetration into cortex, strongly suggesting the role of fungal effectors that actively alter plant immunity. In addition, the current transcriptome profiling study is more comprehensive than the previous one where an Affymetrix microarray (Affymetrix, Santa Clara, CA, USA) was used, which did not have all the *M. truncatula* genes on it. Nevertheless, both the studies strongly suggest that *P. omnivora* turns down the defence signalling pathways for successful colonization in *M. truncatula*. In contrast to *M. truncatula*, *A. thaliana* and *B. distachyon* were both resistant to varying degrees to the fungal infection in the culture tube assays.

### 4.1 Primary metabolism, *BAK1* signalling pathway, and root cell walls play key roles in *B. distachyon* resistance

Traditional defences involving genes in SA and JA–ET signalling pathways or the NBS–LRR responses were absent in *B. distachyon*. Gene enrichment analysis identified that resistance response in *B. distachyon* to *P. omnivora* corresponded with the active upregulation of carbohydrate metabolism. Genes encoding cellulose synthases, enzymes in glycolysis pathways, and tricarboxylic acid cycle were upregulated at all infection time points. Several studies have indicated that sugars act as signalling molecules during fungal attack. Finger millet leaves with enhanced levels of carbohydrates from plants grown in continuous light or 12‐hr photoperiod cycle had enhanced carbohydrates and enhanced resistance to Helminthosporium tetramera (Vidhyasekaran, 1974). In a more recent study, cold acclimatized grasses accumulated sugars that enhanced their resistance to fungal pathogens (Rapacz, Plażek, & Niemczyk, 2000). The study of vascular wilt pathogen *Fusarium oxysporum* on *Lupinus luteus* grown with and without sucrose indicated that sugars may be involved in resistance mechanism (Morkunas, Marczak, Stachowiak, & Stobiecki, 2005). Trehalose, a non‐reducing disaccharide, provides stress tolerance to plants and enhances activity of peroxidase and phenylalanine ammonia lyase (Govind, Jogaiah, Abdelrahman, Shetty, & Tran, 2016; Muchembled, Sahraoui, Grandmougin‐Ferjani, & Sancholle, 2006; Reignault et al., 2002). Trehalose‐6‐phosphate synthase involved in the trehalose biosynthetic pathway (Bradi3g50810) was upregulated in *B. distachyon* upon *P. omnivora* infection at all time points tested, indicating its potential role in the defence mechanism. Thus, the upregulation of carbohydrate metabolism genes and lack of traditional PTI and ETI responses in *B. distachyon* indicate that sugars may play an important role in resistance against *P. omnivora*.

BAK1 homolog (*Bradi4g18280*) was upregulated from 3 to 10 dpi upon *P. omnivora* infection in *B. distachyon*. BAK1 is an LRR kinase that binds to PAMP receptors and induces defence responses in plants through the activation of mitogen‐activated protein kinase (MPK6) and generation of reactive oxygen species (Chinchilla et al., 2007; Yasuda et al., 2017). The strong resistance response in *B. distachyon* to *P. omnivora* also correlated with the observations that the plants produced enhanced root hair growth during the infection process, thus limiting the fungal access to root epidermis. In some instances, when the fungal hyphae reach the epidermal cells, the intercellular, intracellular, and cortical growth are effectively inhibited (Figure *2*). These results were further supported by an observation that a cell wall‐defective mutant, *Bdwat1*, which failed to induce root hairs, was partially compromised to *P. omnivora* and allowed both intercellular and intracellular penetration in root epidermal cells. The enhanced susceptibility of *Bdwat1* mutant indicates that *B. distachyon* cell walls are resilient to *P. omnivora* infection.

### 4.2 *A. thaliana* launches aggressive defence responses using traditional PTI and ETI pathways

Induction of wound‐responsive DAMPs like PROPEP2 and stress‐responsive genes encoding proteins like sHSPs, glutathione *S*‐transferases, and oxidoreductases at 1 dpi indicates active PTI. TNLs and CNLs, previously shown *(*Sinapidou et al., 2004; Staal, Kaliff, Bohman, & Dixelius, 2006 *)* to trigger effective defence response against fungal pathogens like *P. parasitica* and *Leptosphaeria maculans*, were also upregulated in the *P. omnivora–A. thaliana* interaction as part of ETI response. The induction of these *NBS–LRR* genes coincided with the induction of SA‐dependent defence responsive genes and kinase‐encoding genes. SA defence pathway gene *NDR1* and several mitogen‐activated protein kinase pathway genes that were induced in *P. omnivora–A. thaliana* interaction were implicated in resistance mechanisms to other fungal pathogens (Century et al., 1995; Genenncher et al., 2016; Song et al., 1995; Wang, Song, Ruan, Sideris, & Ronald, 1996).

One of the initial responses of *A. thaliana* to *P. omnivora* infection was the induction of genes encoding sHSPs and regulators of sHSPs. These included *AtHSP90*/*70*/*83*/*81*/*40*. The roles of *AtHSP90*/*70*/*40* in plant immunity were previously described (Hubert et al., 2003; Park & Seo, 2015). The defence mechanism in *A. thaliana* was also characterized by the induction of *WRKY* genes and glucosinolate production genes, both of which were uniquely upregulated in this interaction. *AtWRKY51* represses JA signalling and mediates SA defence signalling response in *A. thaliana* to confer resistance to *Alternaria brassicicola* (Gao, Venugopal, Navarre, & Kachroo, 2011). *AtWRKY19*/33/62/40/5/51 involved in biotic stress responses were induced during *P. omnivora* infection (Cai et al., 2015; Kim, Lai, Fan, & Chen, 2008; Rushton, Somssich, Ringler, & Shen, 2010; Xie, Zhou, Deng, & Guo, 2010; Xu, Chen, Fan, & Chen, 2006).

### 4.3 Disease studies in *A. thaliana* and *Bdwat1* mutant indicate fungal plasticity to adapt to host responses

Our results indicate that *A. thaliana* activates DAMP‐mediated response pathway involving PEP2 by 1 dpi when infected with *P. omnivora*. Although the fungal growth was inhibited and there was no fungal growth along the roots at this state, we observed active defence responses in the plant roots. This indicates that the fungus perhaps secretes toxins and other molecules in an attempt to cause disease. *Atpepr1* and *Atpepr2* mutant studies showed enhanced susceptibility to the pathogen indicating that PEPR‐mediated defences play an important role in the basal immunity response against *P. omnivora* as early as 1 dpi. The PEPR pathway induced basal resistance when microbe‐associated molecular pattern‐triggered defences were compromised during *Colletotrichum higginsianum* infection in *A. thaliana* (Yamada et al., 2016). In our study, SA‐mediated defence pathways were upregulated along with genes encoding JAZ proteins, indicating suppression of JA‐mediated biotic defence pathway from 3 dpi onwards. This corresponded to slow growth of the fungus into the culture tubes as well as intracellular root epidermal colonization. The invasive hyphae did not stain with WGA Alexa 488 unlike the intercellular hyphae. Alexa 488 binds to chitin in cell walls. Plants recognize chitin and induce primary immune responses. In order to evade these responses, pathogenic fungi are known to alter the localization of chitin in their cell walls. Studies in *Magnaporthe grisea* showed that the bulbous invasive hyphae localize chitin further into the cell wall, whereas α‐1,3, glucans and chitosan were localized into the accessible regions of the cell walls as a strategy to circumvent host recognition (Fujikawa et al., 2009). We hypothesize that *P. omnivora* alters chitin localization in a similar manner to evade detection during root epidermal cell penetration in *A. thaliana*.

In the susceptible *M. truncatula*, *P. omnivora* adopts a necrotrophic infection strategy, effectively damaging cells ahead of its infection indicated by apoplastic and ER markers. However, a similar attempt in *A. thaliana* launched an efficient defence response against the *P. omnivora*. In order to evade these defences, *P. omnivora* appeared to alter its infection strategy, characterized by intracellular invasive hyphae that had potentially altered chitin localization in the outer cell walls to avoid recognition by host. In response to the switched fungal infection strategy, the *A. thaliana* induced SA‐mediated defence pathways indicating a transient biotrophic infection stage. Genes expressing CNL proteins regulated by *NDR1* were also induced during this time, further confirming the transient biotrophic phase. Studies in *M. phaseolina* identified a similar infection strategy during charcoal root rot disease in sesame where a nonsymptomatic biotrophic phase correlated to SA‐mediated defences (Chowdhury, Basu, & Kundu, 2017).

Although *P. omnivora* failed to infect *B. distachyon* wild‐type plants, it successfully penetrated root epidermal cells of *Bdwat1* mutant. Similar to *A. thaliana* infection, the intracellular invasive hyphae failed to stain with WGA Alexa 488 in this mutant. The bulbous invasive hyphae grew to fill up the first infected cell in *Bdwat1* and later branched off into thin, long hyphae that penetrated the neighbouring cells. The invasive bulbous hyphae is a characteristic of transient biotrophic phase, and the thin, long hyphae correspond to necrotrophic hyphae (Kankanala, Czymmek, & Valent, 2007). Further characterization of the intracellular invasive hyphae and the gene expression during this interaction will be important to identify key pathogen virulence genes. Thus, our results reveal the existence of two distinct infection modes in *P. omnivora*. Furthermore, the *P. omnivora*–*A. thaliana* interaction indicates fungal plasticity where it potentially alters its infection strategy to evade the initial wound‐induced host defences. This fungal plasticity potentially explains the broad host range for this pathogen and failure to identify any effective resistant alfalfa and cotton cultivars so far. Most importantly, this work identifies an important challenge about dealing with fungal pathogens that exhibit high plasticity by altering their infection strategies. This research indicates exploring novel avenues to accomplish PRR disease control methods in the field. These could involve RNA interference silencing‐based approaches or small peptide‐based approaches.

## CONFLICT OF INTEREST

The authors declare no conflict of interest.

## AUTHOR CONTRIBUTIONS

PK, DAJ, and KSM designed the research. PK performed the research. PK, PJ, and RSN did data analysis. PK, RSN, PJ, and KSM wrote the manuscript. All authors read and approved the manuscript.

## Supporting information


**Figure S1** Culture tube assay of *P. omnivora* (*Po*) infection.
**Figure S2.** Illustration of disease progression and infection strategies employed by *P. omnivora* in three plant species ‐ *M. truncatula*, *B. distachyon* and *A. thaliana*.
**Figure S3.** RT‐qPCR validation of *M. truncatula* jasmonate and defence signaling pathway genes, *Ethylene Responsive Factor –1b* (*ERF‐1b*), *Chalcone Synthase* (*CHS*), *Chitinase (CHI)*, *Isoflavone Reductase* (*IFR*), *Lipoxygenase* (*LOX*), *Cysteine Rich Receptor Like Kinase* (*RLK*) and *Allene Oxide Synthase* (*AOS*) at 3 dpi. Bars represent mean of relative fold change from three biological replicates compared to control (0 dpi). Comparison of means was done using student's *t*‐test. ** *p* value <0.01, *** *p* value <0.001.
**Figure S4.** Mapman illustration of biotic stress responses in the three plant species at 1dpi, 5 dpi and 10 dpi
**Table S1.** Primer sequences used for RT‐qPCRs
**Table S2.** Summary of transcriptional data analysis
**Table S3** Infection biology features during *P. omnivora* infection in three plant species
**Table S4.** Percentages of two fold and above DEGs in the three plant species
**Table S5a‐b.** GO enrichment Analysis of *M. truncatula* upregulated and downregulated DEGs **Table S6a‐b.** GO enrichment Analysis of *B. distachyon* upregulated and downregulated DEGs **Table S7a‐b.** GO enrichment Analysis of *A. thaliana* upregulated and downregulated DEGs **Table S8a‐b.** Comparative GO enrichment of upregulated and downregulated DEGs in three plant species
**Table S9.** Gene Enrichment analysis at early and late infection stages
**Table S10.** List of protein classes involved in plant immunity
**Table S11**. Defense related genes in the upregulated DEGs in three plant species

**Dataset S1** DEGs at each infection time point in *M. truncatula* in comparison to control and in comparison to adjacent infection time point.

**Dataset S2** DEGs at each infection time point in *B. distachyon* in comparison to control and in comparison to adjacent infection time point.

**Dataset S3** DEGs at each infection time point in *A. thaliana* in comparison to control and in comparison to adjacent infection time point.

**Dataset S4** GO terms significantly over represented in *M. truncatula* DEGs at each infection time point compared to control generated in AgriGO website.

**Dataset S5** GO terms, protein domains and pathways significantly over represented in *M. truncatula* DEGs at each infection time point compared to control generated in Medicmine website.

**Dataset S6** GO terms significantly over represented in *B. distachyon* DEGs at each infection time point compared to control generated in AgriGO website.

**Dataset S7** GO terms significantly over represented in *A. thaliana* DEGs at each infection time point compared to control generated in AgriGO website.

**Dataset S8** GO terms, protein domains and pathways significantly over represented in *A. thaliana* DEGs at each infection time point compared to control generated in Thalemine website.

**Dataset S9** Gene enrichments in *M. truncatula* DEGs at early and late infection time points

**Dataset S10** Gene enrichments in *B. distachyon* DEGs at early and late infection time points

**Dataset S11** Gene enrichments in *A. thaliana* DEGs at early and late infection time points

**Dataset S12** Positively co‐regulated genes in two fold and above *M. truncatula* DEGs at early and late infection time points.

**Dataset S13** Positively co‐regulated genes in two fold and above *B. distachyon* DEGs at early and late infection time points.

**Dataset S14** Positively co‐regulated genes in two fold and above *A. thaliana* DEGs at early and late infection time points.
